# Health and Sexual Health Care-Seeking Behaviours of Culturally and Linguistically Diverse Men in Australia: Findings From the Ten to Men Longitudinal Study

**DOI:** 10.1177/15579883261438785

**Published:** 2026-05-12

**Authors:** Patience Castleton, Zelalem Mengesha, Mumtaz Begum, Zohra S. Lassi

**Affiliations:** 1 School of Public Health, College of Health, Adelaide University, Adelaide, South Australia, Australia; 2 Robinson Research Institute, Adelaide University, Adelaide, South Australia, Australia; 3 Health Research Institute, University of Canberra, Canberra, Australian Capital Territory, Australia; 4 Adelaide Medical School, College of Health, Adelaide University, Adelaide, South Australia, Australia

**Keywords:** migrant health, refugee health, sexual and reproductive health, CALD, men’s health

## Abstract

Men represent more than half of the world’s migrant population, yet their health care challenges remain under researched. Evidence highlights that culturally and linguistically diverse (CALD) men face substantial barriers to health care access post-migration, particularly in sexual and reproductive health (SRH). This study aimed to analyse health care access, including SRH, among Australian-born and CALD men, using data from the *Ten to Men* Longitudinal study, collected across four waves (2013–2022), including 12,661 non-CALD and 1,106 CALD men. Cross-sectional analyses of data from the first and last waves compared access to SRH and health care between these men, with longitudinal analyses conducted for outcomes measured across multiple waves. Overall, CALD men were less likely to have accessed health care in the last 12 months compared with non-CALD men, including specialist, allied and mental health care. CALD men were more likely to encounter socio-economic (odds ratio [OR]: 1.17; 95% confidence interval [CI]: 0.69, 1.97) and COVID-19 barriers (2.01; 95% CI: 1.22, 3.34) when accessing health care. Similar patterns were observed in SRH access, where CALD men were less likely to seek medical help (OR: 0.59; 95% CI: 0.28, 1.21) than non-CALD men, consistent with longitudinal findings. CALD men were less likely to seek health care or seek medical professionals for SRH and were more likely to face socio-economic barriers than non-CALD men. The findings underscore the urgent need for increased accessibility to culturally sensitive health care immediately post-migration. Tailoring information and services to diverse languages and cultural needs, alongside policy reforms addressing socio-cultural barriers, is essential to ensure equitable and inclusive health care systems.

## Background

The 2020–2030 Australian National Men’s Health Strategy acknowledges the limited research and understanding of the health care needs of men from culturally and linguistically diverse (CALD) backgrounds and calls for greater research action in this space (Commonwealth of Australia, Department of Health, 2019). CALD is a term commonly used in Australian contexts to represent individuals born overseas or who are recent descendants of migrants from diverse language groups and cultures; however, the definition is not used consistently. The Australian Bureau of Statistics’s (ABS, 2022b) definition of CALD is a person who is of Non-Aboriginal and/or Torres Strait Islander descent and speaks a language other than English at home representing a significant portion of the Australian population. Globally, men represent approximately 52% of the world’s 304 million migrants (International Organization for Migration [IOM], 2024). In Australia, where migrants comprise over 30% of the population (Australian Bureau of Statistics [ABS], 2025), CALD men play a significant, yet often overlooked, part of the national health landscape. Migrant and refugee men globally exhibit a range of poor health outcomes, including nutritional deficiencies, incomplete vaccinations, and increased vulnerability to sexual and reproductive health (SRH) issues such as higher risk of HIV, sexual difficulties, and sexually transmitted infections (STIs) (Marukutira et al., 2020; Mengesha et al., 2025; Tiong et al., 2006). Delayed or limited access to timely preventive care can lead to higher hospitalisation rates and increased financial burden (Department of Health, 2021). Recognising these challenges, the Australian National Men’s Health Strategy specifically identifies CALD men as a priority group for SRH interventions, due to both their heightened risk of poor health and the significant barriers they face in accessing appropriate care services.

Globally, men are significantly less likely than women to engage in preventive health services, adhere to treatment plans, or seek timely medical care, which can contribute to higher disease burden and mortality (Feraldi et al., 2025). In Australia, this trend persists, with recent data from the Australian Bureau of Statistics (ABS, 2020c, 2024) showing that, across a 12-month window, women are more likely than men to visit general practitioners (GPs) (87.3% vs. 77.7%), medical specialists (42.7% vs. 35.6%), and mental health professionals (21.6% vs. 12.9%, respectively).

These gender disparities are particularly pronounced in the context of SRH, where men, especially from CALD backgrounds, are less likely than women to seek professional advice or services (Kong et al., 2011; Morgan & Haar, 2008). For example, recent Australian data showed that men had lower annual numbers of GP consultations compared with their female counterparts (4.6% vs. 6.1%) (Bayram et al., 2016). Among young CALD men, fears of judgement or shame from health care professionals and family members further deter engagement with SRH services (Botfield, Newman, et al., 2018). A recent systematic review that included 38 studies focusing on STI and HIV testing among refugees in high-income countries, including Australia, revealed that over half of the participants had never been tested (Santoso et al., 2022). In addition, another scoping review found that migration brought a perceived loss of power and new sexual opportunities to men, influencing the ways they cared for their SRH (Castleton et al., 2025). Reported barriers included financial constraints, transport difficulties, and a lack of knowledge about SRH, underscoring the urgent need for targeted and culturally responsive interventions (Castleton et al., 2025; Santoso et al., 2022).

In Australia, many men from CALD backgrounds report having both inaccurate (Baker et al., 2022) and inadequate (Migrant and Refugee Women’s Health Partnership, 2019) understanding of their own SRH and related conditions, often due to a lack of comprehensive SRH education in their countries of origin (Agu et al., 2016). This low SRH literacy hinders their ability to recognise health concerns, seek appropriate care, and communicate openly about SRH issues, contributing to stigma, shame, and anxiety around the topic (Aibangbee et al., 2024; Schnitzler et al., 2023). The longer this deterrence from health services occurs, the more prominent these barriers become, leading to further delays in diagnosis and treatment. A delayed response to SRH conditions prevents early detection and timely intervention, often resulting in more complex treatment needs and poorer prognoses (Malek et al., 2013; Ziersch et al., 2021). Engaging men in their own SRH is essential not only in improving their health but also the health of partners and children (Agustina et al., 2024; Roudsari et al., 2023). While battling through masculine characteristics and gender norms that position SRH as a feminine issue is challenging, men play an important role in preconception health and must be better included in all SRH services and programmes. Men have previously reported unfavourable environments, and a lack of targeting to men in health centres dissuades them from attending SRH services, even with their wives (Roudsari et al., 2023). Advancing in SRH equality between genders and engaging men in family planning and SRH care is vital in advocating for equal SRH rights.

Due to significant gender disparities in health outcomes, Australia is one of the four countries in the world to have a dedicated men’s health policy, highlighting the country’s commitment to advancing men’s health and well-being (Baker & Shand, 2017). Traditionally, men are marginalised in SRH services and programme development, making their specific needs continually hidden and unaddressed. A 2022 Australian Government-mandated review highlighted that research into the health care access barriers and opportunities for CALD men remains minimal compared with other population groups (Macdonald et al., 2022), limiting our understanding of their interaction with the health care services. Data specific to their experiences with specialist services, including SRH care, is even more limited (Mengesha et al., 2023). Moreover, trends in health care service utilisation, including frequency, reasons, and outcomes of SRH-related health care-seeking, are poorly documented among CALD men (Mengesha et al., 2023), constraining efforts to design responsive and inclusive health care strategies.

This study addresses these critical gaps by analysing data from the Australian Longitudinal Study on Male Health (*Ten to Men*), with the aim of comparing SRH-related health care-seeking behaviours between CALD and non-CALD men in Australia over a 10-year period. In addition, the study explores the barriers faced by both groups in accessing SRH and other health care services. Understanding these trends and barriers is essential for informing culturally sensitive health care delivery and ensuring that health promotion and services are responsive to the needs of CALD communities.

## Methods

### Data Source and Participants

**Figure 1.**
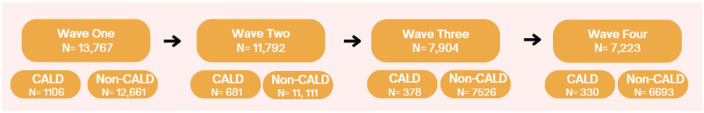
Flow diagram showing how many participants were included from each wave of data collection by CALD and non-CALD status.

This longitudinal study utilised data from Ten to Men (2013–2022), a nationally representative Australian research initiative aimed at understanding men’s health. The cohort was recruited using a stratified, multi-stage, cluster random sample design, recruited through fieldworkers who approached households in selected areas (Currier et al., 2016). The database includes deidentified data from nearly 16,000 men aged 10 to 55 years. As shown in Figure 1, data were collected across four waves: Wave 1 (2013–2014, *n* = 13,767), Wave 2 (2015–2016, *n* = 11,792), Wave 3 (2020, *n* = 7,904), and Wave 4 (2022, *n* = 7,223) (Currier et al., 2016).

Men were eligible if they were aged between 10 and 55 years, were permanent Australian residents or citizens, and had sufficient proficiency in English to complete the self-administered survey questionnaire. The survey, comprising primarily multiple-choice questions, explored six key domains of men’s health: use of health services, health-related behaviours, well-being and mental health, health knowledge, health status, and social determinants.

For this study, we focused on data from adult men (aged 18 years and above) across all four waves. Specifically, we examined participants’ use of general health care services and SRH services over time. Our key variables of interest included health care utilisation, SRH service use, and the factors influencing these behaviours.

### Outcomes

#### Primary Outcome

The primary outcome of this study was the use of health care services among men, assessed using five survey items from the Ten to Men Study.

1. Health care services accessed in the last 12 months were assessed in Wave 3 only. This item consisted of 26 questions asking men whether they had accessed various types of health care services, including GPs, dentists, acupuncturists, and mental health professionals. Responses were dichotomised (‘accessed’ or ‘did not access’) and grouped into five categories (GPs/nurses, specialists, mental health professionals or allied health professionals, or no services accessed) for analysis.
2. Reasons for not seeking health care were captured using 14 questions included across all four waves. These questions addressed a range of barriers, socio-economic, financial, transport, language, personal, and structural, to accessing health care in Australia. For analysis, responses were categorised into four thematic groups. Questions related to COVID-19-specific barriers were included only in Waves 3 and 4, reflecting the pandemic context.
3. The first health information source was assessed in Wave 1 through a set of nine questions asking participants where they typically first seek health-related information. Response options included friends, family, doctors, and online resources. These were classified into four categories for analysis.
4. Preference for a male doctor was assessed in Wave 1 via a single question that asked participants whether they ‘always’, ‘for certain things’, or ‘do not’ prefer a male doctor.
5. Concern about personal health was also assessed in Wave 1 through a binary (yes/no) question: ‘I worry about my health’. This item was included as a proxy for health care–seeking attitudes.

#### Secondary Outcome

The secondary outcome, access to SRH care, was measured using three items from Waves 2, 3, and 4 of the survey.

1. Use of SRH services in the last 12 months was assessed in all three waves by asking participants whether they had used any SRH-related services in the past 12 months. Options included medical doctors, psychologists, and self-help books. Responses were categorised into four groups for analysis.
2. Access to SRH infection testing was assessed in Waves 3 and 4, where men were asked whether they had undergone testing for HIV, chlamydia, gonorrhoea, or syphilis in the past 12 months. This was treated as a binary (yes/no) variable.
3. Visit to sexual health clinic was assessed as a single binary outcome in Waves 2, 3, and 4, asking whether participants had visited a sexual health clinic in the past 12 months.

### Exposure Variables

The exposure groups, CALD and non-CALD, were defined using the Australian Bureau of Statistics (ABS, 2022b) classification of CALD populations and country of birth (COB). Following ABS guidelines, individuals were classified as CALD if they reported a main language other than English spoken at home and were of non-Indigenous background (ABS., 2022b).

### Confounding

Potential confounders of the association between CALD status and access to general and sexual and reproductive health care (SRH) services were identified prior to analysis through a review of relevant literature and the construction of a directed acyclic graph (DAG) (Supplemental Figure 1). Based on this process, we adjusted for a set of confounding variables known to influence health care-seeking behaviours, which may differ systematically between CALD and non-CALD men.

These included access to private health insurance, geographic location, disability status, employment status, education level, and COB.

1. Private health insurance (not including Medicare) was assessed as a binary variable across all four waves.
2. Country of birth was collected in each wave and originally recorded as specific countries and continents. For analysis, this was recoded into a binary variable: ‘within Australia’ versus ‘outside of Australia’.
3. Geographic location in Australia was categorised based on the Australian Statistical Geography Standard (ASGS) region in all four waves as ‘major city’, ‘regional’, or ‘remote’.
4. Disability status was recorded as a binary outcome (‘with disability’ or ‘without disability’) in all four waves.
5. Highest education level was assessed in each wave and categorised into: ‘no education after high school’, ‘apprenticeship/TAFE/trade’, ‘university diploma or degree’, and ‘master’s or postgraduate degree’.
6. Employment status was only collected in Waves 3 and 4, with responses categorised as: ‘employed/working for profit or pay’, ‘unemployed and looking for work’, or ‘neither working nor looking for work’. Given its potential to change over time, employment status was treated as a time-variant variable and therefore could only be adjusted in analyses of data from the waves where this information was recorded.

### Statistical Analysis

We summarised the demographic characteristics of study participants from each wave using frequencies and percentages for categorical variables. First, in a repeated cross-sectional analysis of Wave 1 and Wave 4, we estimated both crude and adjusted associations between CALD status and access to general and SRH health care services in Australia. Depending on the nature of the outcome variables, we employed either binary logistic regression, presented as odds ratio (OR), or multinomial logistic regression, presented as risk ratio (RRR) with 95% confidence intervals (CIs). Outcomes that were grouped into groups for analysis as specified above, such as ‘First health information source’ and ‘Reasons for not seeking health care’, were analysed as binary outcomes to ensure that those who selected multiple responses were not overlooked in one response over another. Wave 1 was selected due to its larger sample size, offering greater statistical power, while Wave 4 provided the most recent data, capturing current patterns in health care access.

Second, for outcomes measured across multiple waves, we conducted longitudinal analyses using random effects multinomial logistic regression models. To determine whether fixed or random-effects models were more appropriate for each outcome, we applied the Hausman test. The test results indicated that random-effects models were the best fit for all eligible outcomes. Accordingly, random-effects models were applied to all primary and secondary outcomes available/measured in more than one wave.

All data management and statistical analyses were conducted using Stata version 17 (StataCorp., 2025). Results were interpreted in line with the American Statistical Association’s (ASA) guidelines on statistical significance and *p*-values (Yaddanapudi, 2016).

## Results

Across all waves of the Ten to Men study, 1,106 men were classified as CALD and 12,661 as non-CALD.

Table 1 presents the background characteristics of the study participants included in the analysis. The distribution of variables was similar across the waves for both CALD and non-CALD men; however, some differences emerged between the two groups.

**Table 1.** Socio-Demographic Characteristics of Participants.

Variables	Wave 1—2013 *N* (%)		Wave 2—2016 *N* (%)		Wave 3—2020 *N* (%)		Wave 4—2022 *N* (%)	
	Non-CALD	CALD	Non-CALD	CALD	Non-CALD	CALD	Non-CALD	CALD
	12661 (91.97)	1106 (8.03)	11111 (94.22)	681 (5.77)	7526 (95.22)	378 (4.78)	6693 (95.30)	330 (4.70)
**Age of participants**								
18-30 years	3432 (27.1)	290 (26.2)	2265 (20.4)	114 (16.7)	1387 (18.4)	33 (8.7)	1123 (16.8)	25 (7.6)
31-40 years	3294 (26.0)	431 (39.0)	2380 (21.4)	260 (38.2)	1224 (16.3)	115 (30.4)	1056 (15.8)	76 (23.0)
41-50 years	4040 (31.9)	270 (24.4)	3150 (28.4)	197 (28.9)	1989 (26.4)	136 (36.0)	1622 (24.2)	128 (38.8)
51+ years	1895 (15.0)	115 (10.4)	2224 (20.0)	98 (14.4)	2673 (35.5)	86 (22.8)	2656 (39.7)	95 (28.8)
**Private health insurance status**								
With private health insurance	6625 (52.3)	480 (43.4)	5672 (51.0)	349 (51.2)	4289 (57.0)	170 (45.0)	3712 (55.5)	155 (47.0)
Without private health insurance	5535 (43.7)	553 (50.0)	4037 (36.3)	301 (44.2)	2758 (36.6)	170 (45.0)	896 (13.4)	41 (12.4)
Missing data	501 (4.0)	73 (6.6)	1402 (12.6)	31 (4.6)	479 (6.4)	38 (10.1)	2085 (31.2)	134 (40.6)
**Disability status**								
With Disability	891 (7.0)	53 (4.8)	813 (7.3)	40 (5.9)	469 (6.2)	12 (3.2)	429 (6.4)	11 (3.3)
Without Disability	11590 (91.5)	1036 (93.7)	9623 (86.6)	623 (91.5)	7020 (93.3)	362 (95.8)	6236 (93.2)	319 (96.7)
**Sexuality**								
Heterosexual	11586 (91.5)	777 (70.3)	526 (4.7)	3 (0.4)	6771 (90.0)	275 (72.8)	5513 (82.4)	241 (73.0)
Homosexual or Bisexual	349 (2.8)	64 (5.8)	27 (0.2)	0 (0.0)	420 (5.6)	32 (8.5)	330 (4.9)	12 (3.6)
Not sure	187 (1.5)	72 (6.5)	11 (0.1)	1 (0.1)	45 (0.6)	26 (6.9)	28 (0.4)	5 (1.5)
Other^a^	140 (1.1)	66 (6.0)	1 (<1)	1 (0.1)	33 (0.4)	4 (1.1)	34 (0.5)	1 (0.3)
**Relationship Status**								
Single or separated^b^	Not asked		432 (3.9)	4 (0.6)	1508 (20.0)	42 (11.1)	1274 (19.0)	34 (10.3)
In a relationship^c^			131 (1.2)	0 (0.0)	5774 (76.7)	313 (82.8)	5267 (78.7)	288 (87.3)
**Highest education received**								
No Education after high school^d^	3205 (25.3)	183 (16.5)	2388 (21.5)	91 (13.4)	Not asked		1582 (23.6)	30 (9.1)
Apprenticeship, Trade, TAFE Certificate^e^	5341 (42.2)	186 (16.8)	4223 (38.0)	130 (19.1)	2056 (27.3)	35 (9.3)	1672 (25.0)	25 (7.6)
University Diploma or Degree	2106 (16.6)	357 (32.3)	1807 (16.3)	224 (32.9)	2521 (33.5)	142 (37.6)	2478 (37.0)	141 (42.7)
Master’s Degree, Postgraduate Degree or Postgraduate Diploma	959 (7.6)	278 (25.1)	868 (7.8)	189 (27.8)	655 (8.7)	100 (26.5)	725 (10.8)	112 (33.9)
Other	252 (2.0)	9 (0.8)	236 (2.1)	4 (0.6)	131 (1.7)	3 (0.8)	36 (0.5)	6 (1.8)
**Employment status**								
Currently employed^f^	Not asked		Not asked		6282 (83.5)	312 (82.5)	5764 (86.1)	296 (89.7)
Not employed					925 (12.3)	37 (9.8)	749 (11.2)	17 (5.2)
**Country of birth**								
Australia	10587 (83.6)	0 (0.0)	6004 (54.0)	2 (0.3)	6535 (86.8)	9 (2.4)	3971 (59.3)	1 (0.3)
Outside Australia	2048 (16.2)	1103 (99.7)	939 (8.5)	411 (60.4)	955 (12.7)	354 (93.7)	670 (10.0)	221 (67.0)
**Self-rated Health**								
Very good	Not asked		Not asked		927 (12.3)	71 (18.8)	698 (10.4)	39 (11.8)
Good					2918 (38.8)	145 (38.4)	2467 (36.9)	142 (43.0)
Fair/poor					2653 (35.3)	125 (33.1)	2551 (38.1)	126 (38.2)
**Region**								
Remote	Not asked		14 (0.1)	0 (0.0)	26 (0.3)	1 (0.3)	28 (0.4)	1 (0.3)
Regional	5521 (43.6)	146 (13.2)	4664 (42.0)	85 (12.5)	2756 (36.6)	30 (7.9)	2515 (37.6)	29 (8.8)
Major cities	7138 (56.4)	960 (86.8)	6362 (57.3)	596 (87.5)	4295 (57.1)	327 (86.5)	3915 (58.5)	287 (87.0)

a Pansexual, Queer, Asexual, use a different pronoun.

b Single, Divorced, separated, widowed.

c In a relationship, living with a partner, engaged, married.

d Have not completed any additional qualifications, begun but have not completed any qualifications.

e Including certificate II/IV and I/II.

f Full-time, part-time, self-employed and unpaid work for a family business.

In Wave 1, non-CALD men were more likely to be living in regional areas (43.6% vs. 13.2%) and to be over 51 years of age (15.0% vs. 10.4%) compared with CALD men, findings consistent throughout all collection waves. Educational attainment also differed between the groups in the first wave: a higher proportion of non-CALD men had completed an apprenticeship or Technical and Further Education (TAFE) certificate (42.2% vs. 16.8%), while fewer had completed a master’s or postgraduate degree (7.6% vs. 21.5%). Interestingly, these gaps slightly shifted throughout the subsequent waves, with Wave 4 seeing a narrower gap in TAFE qualifications (25.0% vs. 7.6%), and a wider gap in postgraduate qualifications (10.8% vs. 33.9%) between non-CALD and CALD men.

Employment status was collected in the final two waves only, with 83.5% of non-CALD men reporting current employment and 82.5% of CALD men reporting employment, with similar patterns seen in the final wave (86.1% vs. 89.7%).

Importantly, only 43.4% of CALD men in Wave 1 and 47.0% in Wave 4 reported having private health insurance, whereas 52.3% of non-CALD men in Wave 1 and 55.5% in Wave 4 had access to this insurance. The majority of the study participants identified as heterosexual; however, more CALD men self-reported as ‘homosexual or bisexual’ (5.8% vs. 2.8%) or ‘not sure’ (6.5% vs. 1.5%) than non-CALD men in Wave 1. These patterns remained consistent throughout all waves; however, in wave 4, slightly more non-CALD men self-reported as ‘homosexual or bisexual’ compared with CALD men (4.9% vs. 3.6%). Small differences were observed between CALD and non-CALD men in terms of self-rated health status and living with disability.

### Use of Health Care Services

CALD men were slightly more likely to have socio-economic barriers and COVID-19 barriers as a reason for not seeking health care than non-CALD men (Table 2). CALD men were more likely to visit GPs/nurses (46.0% vs. 35.8%) and were less likely to visit specialists, allied health professionals, and mental health professionals than non-CALD men. For example, 2.4% of CALD men visited a mental health professional in the last 12 months compared with 5.8% of non-CALD men. Wave 1 showed that the first source of health information for both CALD and non-CALD men was friends and family (45.4% and 49.2%, respectively). Wave 1 also showed that CALD men were more likely to always prefer a male doctor (12.1% and 7.6%, respectively) and were more likely to worry about their health (52.7% and 41.0%, respectively).

**Table 2.** Access to General Health Care Services of CALD and Non-CALD Men in Wave 1.*

Outcome	Non-CALD *N* (%)	CALD *N* (%)	Unadjusted OR ^f^ (95% CI) CALD vs non-CALD	Adjusted OR ^f^ (95% CI) CALD vs non-CALD
Health care services accessed in the last 12 months**	*N* = **7526**	*N* = **378**		
General practitioners/nurses^a^	2697 (35.8)	174 (46.0)	0.68 (0.47, 0.98)	0.28 (0.08, 0.95)
Specialists^b^	1218 (16.2)	41 (10.8)	0.63 (0.45, 0.88)	0.67 (0.46, 0.97)
Mental health professionals^c^	436 (5.8)	9 (2.4)	0.40 (0.20, 0.77)	0.53 (0.26, 1.09)
Allied health professionals^d^	1958 (26.0)	70 (18.5)	0.65 (0.50, 0.84)	0.71 (0.53, 0.97)
No service accessed	829 (11.0)	47 (12.4)	Reference	Reference
			Unadjusted RRR^e^ (95% CI) CALD vs non-CALD	Adjusted RRR^e^ (95% CI) CALD vs non-CALD
**First health information source**	*N* = **12661**	*N* = **1106**		
Doctor	2908 (23.0)	248 (22.4)	1.06 (0.90, 1.24)	0.97 (0.80, 1.17)
Internet	2362 (18.7)	240 (21.7)	1.26 (1.07, 1.48)	0.91 (0.75, 1.10)
Does not seek help or has never had concerns	693 (5.5)	51 (4.6)	0.91 (0.68, 1.23)	1.02 (0.71, 1.46)
Friends, family and other^f^	6226 (49.2)	502 (45.4)	Reference	Reference
**Preference of a male doctor**	*N* = **12661**	*N* = **1106**		
Don’t care	9294 (73.4)	719 (65.0)	0.56 (0.43, 0.73)	1.11 (0.51, 2.42)
Yes, always	963 (7.6)	134 (12.1)	1.00 (0.73, 1.38)	2.01 (0.90, 4.52)
Yes, for certain things	1651 (13.0)	168 (15.2)	0.73 (0.54, 1.00)	1.55 (0.69, 3.45)
No, prefer a female doctor	275 (2.2)	16 (1.4)	0.42 (0.24, 0.74)	0.85 (0.33, 2.25)
Refused to answer	478 (3.8)	69 (6.3)	Reference	Reference

* All outcomes measured in Wave 1 except ** which is measured in Wave 3.

a GP, community nurse, Nurse, GP checkup.

b Aboriginal Health Professional, Dentist, Specialist doctor, Specialist doctor, Cardiologist, Dermatologist, Urologist.

c Accredited counsellor, Alcohol and drug worker, Psychiatrist, Psychologist.

d Acupuncturist, Audiologist, Pharmacist, Chiropodist/Podiatrist, Chiropractor, Dietician, Massage Therapist, Naturopath, Osteopath, Physiotherapist.

e The unadjusted and adjusted results are based on the data from the wave in which they were first measured.

f Friend, partner, family, parents, family member who’s not parents, other.

In the primary cross-sectional analyses adjusted for confounds (Table 2), men from CALD backgrounds were, overall, less likely to have visited a health care service in the last 12 months compared with non-CALD men. Although only asked in the first wave, the likelihood of visiting a specialist in the last 12 months was almost 30% lower among CALD men compared with non-CALD men (OR: 0.67; 95% CI: 0.46, 0.97). CALD men were also 53% less likely to have seen a mental health professional (OR: 0.53; 95% CI: 0.26, 1.09). CALD men were also twice as likely to always prefer seeing a male doctor (RRR: 2.01; 95% CI: 0.90, 4.52).

Following adjustment for confounding, CALD men had an increased risk of experiencing socio-economic barriers in accessing health care services compared to non-CALD men across the longitudinal data collection (Table 3) (OR: 1.17; 95% CI: 0.69, 1.97). However, due to small numbers, the estimate is imprecise and has wide confidence intervals (95% CI: 0.69, 1.97), ranging from 31% lower risk to almost two times higher risk of experiencing socio-economic barriers in accessing health care.

**Table 3.** Reasons for not Accessing General Health Care Services Among CALD Men Compared With Non-CALD Men.

Outcome	Wave 1/2013 *N* (%)		Wave 2/2016 *N* (%)		Wave 3/2020 *N* (%)		Wave 4/2022 *N* (%)		Unadjusted OR^a^ (95% CI) CALD vs. non-CALD	Adjusted OR^a^ (95% CI)^b^ CALD vs. non-CALD
	Non-CALD	CALD	Non-CALD	CALD	Not CALD	CALD	Not CALD	CALD		
	12661 (91.97)	1106 (8.03)	11111 (94.22)	681 (5.77)	7526 (95.22)	378 (4.78)	6693 (95.30)	330 (4.70)		
**Reasons for not seeking healthcare**										
Socio-economic barriers^c^	320 (2.5)	35 (3.2)	255 (2.3)	25 (3.7)	229 (3.0)	8 (2.1)	204 (3.0)	12 (3.6)	1.22 (0.96, 1.53)	1.17 (0.69, 1.97)
COVID-19 barriers^d^	Not asked		Not asked		120 (1.6)	12 (3.2)	172 (2.6)	13 (3.9)	1.31 (0.87, 1.97)	2.01 (1.22, 3.34)
Service barriers^e^	84 (0.7)	2 (0.2)	45 (0.4)	0 (0.0)	148 (2.0)	4 (1.1)	381 (5.7)	9 (2.7)	0.34 (0.51, 0.57)	0.85 (0.46, 1.57)
Other priorities^f^	237 (1.9)	18 (1.6)	153 (1.4)	14 (2.1)	963 (12.8)	33 (8.7)	864 (12.9)	31 (9.4)	0.65 (0.53, 0.79)	0.81 (0.61, 1.08)

a The ORs are based on longitudinal analyses using random-effect binary logistic regression.

b Adjusted for country of birth, employment status, access to private health insurance, and highest education level.

c Not a resident, high costs, transport problems, language problems.

d COVID-19 unvaccinated, not able to leave the house due to COVID-19.

e Not taking on new patients, no skilled doctors, other reasons.

f Decided not to seek care, too busy/other responsibilities, work commitments, busy at work.

CALD men were twice as likely to report health care–related COVID-19 barriers compared with non-CALD men (OR: 2.01; 95% CI: 1.22, 3.34) after adjusting for confounders; however, the estimates were imprecise with wide confidence intervals. Looking at the longitudinal data (Table 3), the random-effects binary logit models show that CALD men are 15% less likely to experience service barriers compared with non-CALD men and almost 20% less likely to have missed a health care opportunity due to competing priorities. However, the estimates for socio-economic barriers were imprecise due to small numbers.

### Use of SRH Care Services

As shown in Table 4, the most reported SRH services used in the second wave of data collection were the internet for CALD and non-CALD men (19.1% vs. 18.7%), with consistent patterns seen in the final two waves of data collection. Collected only in the final two waves, a slightly higher proportion of CALD men reported accessing HIV testing compared with non-CALD men in both Waves 3 and 4 (1.6% vs. 0.7% and 1.2% vs. 0.8%, respectively); however, testing for other STI infection testing (chlamydia, gonorrhoea, and syphilis) within the same two waves was lower among CALD men (0.5% vs. 3.6% and 0.9% vs. 3.3%, respectively). The proportion of men who had attended a SRH clinic in the last 12 months was slightly inconsistent across the three waves in which it was collected, with slightly fewer CALD men reporting visiting SRH clinics in the past 12 months in Waves 2 and 3 (84.8% vs. 84.7% and 95.5% vs. 96.0%, respectively), but more CALD men reporting using these services in Wave 4 (86.4% vs. 77.9%). However, the sample numbers for this outcome are minimal in all three waves of data collection and thus must be interpreted with caution.

**Table 4.** Access to Sexual and Reproductive Health (SRH) Services of CALD and Non-CALD Men in Each Study Wave.^a^.

Outcome	Wave 2/2016 *N* (%)		Wave 3/2020 *N* (%)		Wave 4/2022 *N* (%)	
	Not CALD	CALD	Not CALD	CALD	Not CALD	CALD
	11111 (94.22)	681 (5.77)	7526 (95.22)	378 (4.78)	6693 (95.30)	330 (4.70)
**SRH services used in the last 12 months**						
Medical Doctor^b^	Not asked		493 (6.6)	13 (3.4)	409 (6.1)	11 (3.3)
Non-medical help^c^	73 (0.7)	0 (0.0)	242 (3.2)	16 (4.2)	197 (2.9)	8 (2.4)
Internet	2079 (18.7)	130 (19.1)	653 (8.7)	27 (7.1)	1183 (17.7)	86 (26.1)
No help sought	8959 (80.6)	551 (80.9)	6138 (81.6)	322 (85.2)	4904 (73.3)	225 (68.2)
**Have you accessed SRH infection testing in the last 12 months?**						
HIV	Not asked		51 (0.7)	6 (1.6)	52 (0.8)	4 (1.2)
Other infections (Chlamydia, gonorrhoea and syphilis)			270 (3.6)	2 (0.5)	223 (3.3)	3 (0.9)
None			7165 (95.2)	364 (96.3)	6386 (95.4)	319 (96.7)
**Sexual help-seeking: Sexual Health clinic**						
No	9417 (84.8)	577 (84.7)	7189 (95.5)	363 (96.0)	5783 (86.4)	257 (77.9)
Yes	58 (0.5)	7 (1.0)	80 (1.1)	3 (0.8)	66 (1.0)	0 (0.0)

a SRH Information not asked in Wave 1

b Family doctor, sexual health clinic, psychologist, other clinic or doctor.

c Self-help book, self-help group, relationship counsellor, family or friend.

In the analysis shown in Table 5, CALD men were more likely to seek SRH help from the internet, for example, Wave 2 results showed that CALD men were 1.83 times more likely to seek SRH help from the internet, compared with non-CALD men (OR: 1.8302; 95% CI: 1.39, 2.40), recent data from the fourth wave also showed similar patterns (OR: 1.35; 95% CI: 0.97, 1.88). In the final wave of data collection, CALD men reported being 41% less likely to seek help from a medical doctor for SRH problems (OR: 0.59; 95% CI: 0.28, 1.21), and 29% less likely to engage in non-medical help for their SRH care compared with non-CALD men (OR: 0.71; 95% CI: 0.33, 1.54). Due to limited sample populations, estimates could not be made for previous waves; however, data presented in Table 4 indicate consistent patterns throughout all collection waves. Furthermore, the wide confidence intervals in both outcomes, potentially due to the smaller sample size in the final wave, suggest an impression.

**Table 5.** Use of Sexual and Reproductive Health Service Usage Among CALD and Non-CALD Men in the First and Final Waves in Which the Questions Appeared.

Outcome	CALD vs. non-CALD	The first wave appeared in		The final wave appeared in	
		Unadjusted OR (95% CI)	Adjusted OR (95% CI)^a^	Unadjusted OR (95% CI)	Adjusted OR (95% CI)^b^
**SRH help-seeking** ^c^					
Medical Doctor^d^	Non-CALD	Reference	Reference	Reference	Reference
	CALD	No observations	No observations	0.53 (0.28, 1.01)	0.59 (0.28, 1.21)
Non-medical help^e^	Non-CALD	Reference	Reference	Reference	Reference
	CALD	No observations	No observations	0.63 (0.32, 1.23)	0.71 (0.33, 1.54)
Internet	Non-CALD	Reference	Reference	Reference	Reference
	CALD	1.02 (0.84, 1.25)	1.83 (1.39, 2.40)	1.64 (1.27, 2.12)	1.35 (0.97, 1.88)
No help sought	Non-CALD	Reference	Reference	Reference	Reference
	CALD	1.02 (0.84, 1.24)	0.60 (0.46, 0.78)	0.78 (0.62, 0.99)	0.89 (0.67, 1.19)

a Adjusted for country of birth, access to private health insurance and highest education level.

b Adjusted for country of birth, employment status, access to private health insurance and highest education level.

c Asked in Wave 2 and 4.

d Family doctor, sexual health clinic, psychologist, other clinic or doctor.

e Self-help book, self-help group, relationship counsellor, family or friend.

Reflecting on this data longitudinally, as seen in Supplemental Table 1, there was almost no difference between CALD and non-CALD men in seeking SRH care on the internet (OR: 0.99; 95% CI: 0.66, 1.50). However, CALD men were 46% more likely to seek care from non-medical professionals (OR: 1.46; 95% CI: 0.78, 2.72), although the estimates were imprecise with wide confidence intervals. In addition, Supplemental Table 2 shows that CALD men were 87% more likely to have visited a sexual health clinic in the past 12 months in Wave 2; however, this was not reflected in the longitudinal analysis, which showed they are 42% less likely than non-CALD men to have accessed these services throughout the survey. The sample populations for the cross-sectional analysis were very small and thus need to be interpreted with caution.

Longitudinal analyses (Supplemental Table 1) showed that CALD men were 74% more likely to have received an HIV test (RRR: 1.66; 95% CI: 0.77, 3.59) and 73% less likely to receive another STI test (RRR: 0.27; 95% CI: 0.11, 0.67), compared with non-CALD men. Cross-sectional analysis indicated similar patterns, as seen in Table 5; however, these sample populations were very small and therefore need to be interpreted with caution.

## Discussion

This study used longitudinal data from the Ten to Men study to explore health care and SRH service-seeking behaviours of CALD and non-CALD men. Although over half of the CALD respondents expressed concern about their health, they reported lower engagement with formal health services.

The analysis showed that in Wave 3 (2019–2020), CALD men were 33% less likely than non-CALD men to visit specialists and 47% less likely to seek care from mental health professionals; they were also 28% more likely to have visited a GP or nurse (Table 2). Current literature indicates that the cost of these services is a major contributor to the lack of use among CALD populations, with specialist services, especially those for mental health, more likely to incur out-of-pocket fees (Khatri & Assefa, 2022 & Spike et al., 2011). The number of post-migration stressors that CALD men are under, including employment, housing, and schooling, often takes precedence over health, further deterring the use of essential services (Spike et al., 2011). Despite literature indicating a high prevalence of mental health troubles among CALD migrants in Australia, mental health stigma, service discrimination, affordability, and long waiting lists are among the major contributors to lack of service usage among the group (Fauk et al., 2021).

Low knowledge of health care services beyond GPs, difficulties accessing referral pathways, inadequate health literacy, and communication problems further deter CALD men from accessing specialist services (Au et al., 2019; Khatri & Assefa, 2022). Interestingly, CALD men in the current study were 19% less likely than non-CALD men to report having other priorities that took precedence over health care appointments, such as work commitments and being too busy (Table 3). This finding is contradictory to current literature that indicates that CALD men would be more likely to report barriers such as work commitments for missing health care appointments (Au et al., 2019; Khatri & Assefa, 2022). This variable has not been studied extensively and is an important question to include in future qualitative and quantitative studies that examine CALD men’s relationships with health care and competing priorities. The small sample size of the CALD men in the Ten to Men sample, differences in sample composition, or differences in cultural reporting of reasons for missed care may help to explain this contradictory finding.

Access to the highest attainable physical and mental health, which encompasses the availability and accessibility of acceptable, good quality health care, is a fundamental right (WHO, 2023). In the context of the present study, access to health care refers to an individual’s ability to physically access timely, appropriate, and affordable health care services, including GPs and pharmacies, in-person or via telehealth services.

The current study showed that CALD men are 17% more likely to face socio-economic barriers, including high costs and lack of transportation, when seeking health care compared with non-CALD men, indicating how structural disadvantages disproportionately constrain their health opportunities. These findings are consistent with previous Australian studies (Australian Institute of Health and Welfare, 2024; Khatri & Assefa, 2022), thus urging a clear need for health care systems and policies to adequately meet the needs of diverse communities and break down these structural barriers. Previous studies have shown that CALD men face complex barriers to accessing health care, including difficulties with transportation, language, communication, and costs (Javanparast et al., 2020). Out-of-pocket expenses, particularly for specialist services that are not commonly subsidised, further discourage SRH health care-seeking among CALD men (Javanparast et al., 2020; Macdonald et al., 2022). Interestingly, CALD men in Wave 1 were 66% less likely than non-CALD men to report barriers such as health care services not accepting new patients or a lack of available skilled doctors. By Wave 4, CALD men were only 15% less likely to report these barriers compared with non-CALD men. These changes may be due to the COVID-19 pandemic and changes in health care services during this period; however, the wide CI in the results from Wave 4 shows that the results must be interpreted with caution. While the literature on these barriers is incredibly scarce, existing evidence contradicts these findings, suggesting that CALD populations are more likely than non-CALD populations to report discriminatory health care practices, a shortage of adequate staff, and low acceptability of services in relation to their cultural and religious beliefs (Khatri & Assefa, 2022). Further research with larger and more representative samples is needed to better understand the specific challenges faced by diverse CALD communities when accessing health care, to inform the development of appropriately tailored interventions. A disaggregated collection of health care data could assist in monitoring the extent of structural barriers and ensure accountability and consistency across health care services (Renzaho, 2023).

Notably, CALD men were twice as likely to report experiencing COVID-19-related barriers when accessing any health care service in Wave 3, suggesting that they may have been less able to attend care. The large confidence interval, possibly due to the COVID-19 questions being asked only in Wave 3 (during the pandemic), which had a small sample size, may explain these disparities and thus analysis must be interpreted with caution. According to the ABS, migrants faced a significantly higher risk of COVID-19-related health complications and mortality compared with non-migrants during the pandemic (Australian Bureau of Statistics [ABS], 2022a), which may partly explain both their increased GP attendance and the barriers they encountered during the pandemic. A global study found that migrants in Australia lost more working hours than their Australian-born counterparts during the global pandemic, placing significant strain on both their economic status but also their mental and social well-being (Mengesha et al., 2022). In addition, literature indicates that migrants are more likely to lose their employment first, often due to decreased training levels, shorter years of employment, and increases in discrimination during periods of competitive labour markets (Hintermeier et al., 2024; OECD, 2022). This indicates that greater recognition of CALD communities is needed in pandemic response and social support services during health emergencies, with studies showing these incentives led to better social and health outcomes during the COVID-19 pandemic (Hintermeier et al., 2024).

While literature differentiating CALD men from Australian-born men is scarce, the available evidence, supported by the current findings, suggests that CALD men face unique barriers in accessing SRH care, deterring them from seeking information or care (Agu et al., 2016; Botfield, Zwi, et al., 2018), despite their high risk of poor SRH outcomes (King et al., 2023). It is also important to consider the impact of sexual orientation on men’s access to SRH services. Discrimination and abuse experienced by many gay and bisexual men in Australia, both inside and outside of health care settings, may deter them from seeking care (Lim et al., 2025). Alternatively, the use of HIV prevention medication such as PrEP requires ongoing clinical monitoring, which may facilitate more regular engagement with health care services (Sundareshan et al., 2024).

CALD men in Australia have previously reported being ill-informed about where to seek SRH care, including HIV and STI testing, thus seeking advice from the internet, underscoring the importance of ensuring that credible and informative online resources are accessible (Marukutira et al., 2020). This could result in the misinterpretation of key information or the neglect of seeking further advice and information. It is, therefore, important that future research aims to understand the SRH information preferences of CALD men, ensuring that information is appropriately tailored to a range of languages and cultures. Furthermore, SRH programmes must establish streamlined and easy-to-access services for CALD men, providing them with confidential and culturally sensitive environments for STI and HIV testing.

The current study found that CALD men were likely to prefer visiting a male doctor compared with a female doctor. Current literature is conflicting on the GP gender preferences among CALD and non-CALD men in Australia (Bourke, 2002), and there is limited research that disaggregates by CALD status. However, in many cultures, taboos and limited SRH education shape how men understand and manage their SRH, with many migrants first encountering such topics after migration to Australia (Botfield, Zwi, et al., 2018). This can heavily influence CALD men’s confidence and ability to communicate their needs or concerns with a GP, potentially deterring them from seeking help, especially from a female care provider (Sievert et al., 2018). In addition, more than a quarter (26.1%) of CALD men sought SRH information online before turning to a professional resource in the current study, which could impact the quality and cultural relevance of information accessed. The longitudinal data (Supplemental Table 2) also showed that CALD men were 42% less likely to visit a sexual health clinic compared with non-CALD men. These findings further suggest that CALD men face systemic barriers, including socio-economic disadvantage, that may limit their access to essential and accurate health care, including STI testing. This underscores the importance of offering diverse and culturally sensitive health care services immediately post-migration, including access to multi-cultural services offered in a range of languages. Literature has shown that men from CALD backgrounds prefer to seek SRH advice and information from online and anonymous sources compared with friends, family, or health professionals (Botfield, Zwi, et al., 2018; Mengesha et al., 2023). Negative taboos and cultural stigma established in many CALD men’s home countries have been previously reported to create feelings of shame and embarrassment when discussing SRH, heavily influencing their desires to seek care (Agu et al., 2016; Mengesha et al., 2023). To bridge cultural gaps in understanding, digital health technologies must be up-to-date, culturally responsive, and relevant to the CALD community, allowing easy and cost-free access to health information and care (Matlin et al., 2025). While digital health modalities can reduce reliance on in-person visits and potentially mitigate some access barriers, evidence suggests that CALD communities continue to experience inequalities in telehealth access and digital navigation, often driven by systemic and service-level barriers rather than barriers with technology use itself (Gallegos-Rejas et al., 2023).

Service providers can leverage the SRH care-seeking behaviours of CALD men to promote verified apps and reputable online platforms that provide accurate SRH information. Information packs and resources should be distributed to community centres and made available to migrants and refugees upon entrance to Australia, which provide a clear overview of health care and provide reputable sources of online information.

## Strengths and Limitations

The 10-year longitudinal nature of the Ten to Men study allowed us to examine the changing health care and SRH service utilisation patterns across a significant period. This strengthened our analysis as we were able to examine the data as both cross-sectional and longitudinal, which was especially important due to the low retention of participants across the four waves. ‘The Ten to Men’ study used a stratified, multi-stage, cluster random sampling design to recruit the participants; hence, it is a representative sample of men who are Australian residents and citizens. However, these findings cannot be generalised to non-resident or non-citizen CALD men living in Australia, who could be facing even more barriers in accessing health care due to lack of access to Medicare or subsidised services.

The Ten to Men survey required English language proficiency enough to read and understand each survey question, further highlighting the structural barriers in place for CALD men’s access to, and participation in, health care. This limited the sample pool and resulted in the exclusion of CALD men who lack English proficiency, which may limit the generalisability of the results. This likely led to the underrepresentation of recent arrivals and refugees and those with insecure visa status. It is important to understand that CALD men are not a homogeneous group, and there are variations in health understanding and service use between individuals and communities. However, due to the nature of the survey questions and the small sample population, it was difficult to disaggregate between CALD communities. This may shield important differences between migrant communities, and future research and policies must consider the diversity of the CALD community.

The survey did not collect intersectional information from respondents, such as length of stay in Australia, religion, or access to Medicare, which are likely to influence health service use among CALD men. In addition, many outcomes, including health care services used in 12 months, were not asked in all four waves, largely limiting the sample size for key primary and secondary outcomes, especially if they were not asked in Wave 1, which had the largest sample. However, where possible, cross-sectional analysis of the smaller, later waves still allowed for broader pattern recognition across diverse time periods. The underrepresentation of CALD men in the Ten to Men study limited our ability to accurately interpret many of the outcomes. The small estimates of outcomes, such as rates of HIV testing and socio-economic barriers, were largely imprecise due to limited sample sizes in and across waves. Studies with larger sample populations that better encompass CALD voices are needed to further explore these important SRH areas.

It is also important to acknowledge the definition of CALD is debated and often critiqued as an inappropriate term used to isolate groups of individuals (Pourmarzi et al., 2025). While it is important to recognise and acknowledge this discourse, due to the nature of the data collected in the ‘Ten to Men’ survey, and the use of the term within ABS documentation and data, we chose to use this definition for the study. The ABS definition of CALD used may have influenced the sample size of CALD men included in the study; however, analysis using both CALD status and COB yielded similar results, thus suggesting that the chosen definition of CALD captured the largest proportion of CALD men in the Ten to Men surveys. Finally, as this was a purely quantitative survey, all responses were binary in nature, with no opportunity for respondents to expand upon their responses for more clarity and detail. Survey data was all self-reported, which introduces potential recall biases or biases in social desirability, especially regarding the sensitive SRH topics. Expanding the scope of data collection and integrating a mixed-methods approach to future research could better inform CALD men’s health service usage, assisting in developing more tailored interventions. Furthermore, future research should work to understand gender, culture, language, and health care access through an intersectional lens. Comparing service usage patterns and views of SRH between CALD men and women and exploring differences across CALD communities in different Australian regions would assist in supporting this deeper understanding of disparities in accessing care. Qualitative research is essential for deepening our understanding of CALD men’s experiences of SRH care in Australia. Gaining firsthand insight into the needs and priorities of CALD men when accessing SRH services, as well as the challenges faced by health care providers in delivering SRH care, is important for informing meaningful practice and policy change.

## Conclusions

The current study expanded the current literature on CALD men’s use of health care services in Australia by investigating longitudinal changes and the barriers associated with service access. Our analysis indicated a lower use of mental health and specialist services among CALD men compared with Australian-born men, with CALD men reporting a high risk of socio-economic and COVID-19 barriers. However, the findings also revealed that CALD men reported higher use of GP services, along with greater concern for their health, compared with non-CALD men. This study highlights the need for future research and interventions to critically examine the unique and specific barriers CALD men face in accessing health care, to inform the development of tailored interventions.

## Supplemental Material

sj-docx-1-jmh-10.1177_15579883261438785 – Supplemental material for Health and Sexual Health Care-Seeking Behaviours of Culturally and Linguistically Diverse Men in Australia: Findings From the Ten to Men Longitudinal Study Supplemental material, sj-docx-1-jmh-10.1177_15579883261438785 for Health and Sexual Health Care-Seeking Behaviours of Culturally and Linguistically Diverse Men in Australia: Findings From the Ten to Men Longitudinal Study by Patience Castleton, Zelalem Mengesha, Mumtaz Begum and Zohra S. Lassi in American Journal of Men's Health
