# Supplementary material for: Health and Sexual Health Care-Seeking Behaviours of Culturally and Linguistically Diverse Men in Australia: Findings From the Ten to Men Longitudinal Study
Source: Am J Mens Health. 2026 May 12;20(3):15579883261438785. doi: 10.1177/15579883261438785 (PMC13172675; doi:10.1177/15579883261438785)
Supplement: sj-docx-1-jmh-10.1177_15579883261438785 – Supplemental material for Health and Sexual Health Care-Seeking Behaviours of Culturally and Linguistically Diverse Men in Australia: Findings From the Ten to Men Longitudinal Study [file sj-docx-1-jmh-10.1177_15579883261438785.docx]

***Supplementary Table 1: Regression analysis outcomes for* CALD status and access to sexual and reproductive healthcare services in Australia**

|  | **Unadjusted OR (95% CI)** | | **Adjusted OR (95% CI) ^a^** | |
| --- | --- | --- | --- | --- |
|  | **CALD vs non-CALD** | | |  |
| **SRH services used in the last 12 months** | | | | |
| Medical Doctor ^b^ | Non-CALD | Reference | Reference |  |
|  | CALD | No observations | 0.42 (0.20, 0.89) |  |
| Non-medical help ^c^ | Non-CALD | Reference | Reference |  |
|  | CALD | No observations | 1.46 (0.78, 2.72) |  |
| Internet | Non-CALD | Reference | Reference |  |
|  | CALD | No observations | 0.99 (0.66, 1.50) |  |
| No help sought | Non-CALD | Reference | Reference |  |
|  | CALD | Base outcome | Base outcome |  |
| **Have you accessed SRH infection testing in the last 12 months?** | | | | |
|  | | **Unadjusted RRR (95% CI)** | **Adjusted RRR (95% CI) ^a^** |  |
| HIV | Non-CALD | Reference | Reference |  |
|  | CALD | 1.47 (0.77, 2.81) | 1.66 (0.77, 3.59) |  |
| Other infections (Chlamydia, gonorrhoea and syphilis) | Non-CALD | Reference | Reference |  |
|  | CALD | 0.15 (0.06, 0.37) | 0.27 (0.11, 0.67) |  |
| None | Non-CALD | Reference | Reference |  |
|  | CALD | Base outcome | Base outline |  |

^a^ Adjusted for country of birth, employment status, access to private health insurance, highest education level and region

^b^ Family doctor, Sexual health clinic, Psychologist, other clinic or doctor

^c^ Self-help book, self-help group, relationship counsellor, family or friend

***Supplementary Table 2:* Regression analysis outcomes for use of sexual health clinic service usage amongst CALD and non-CALD men in the first and final waves in which the questions appeared**

| **Have you visited a sexual health clinic in the past 12 months?** | **Unadjusted OR**  **(95% CI)** | **Adjusted OR**  **(95% CI) ^a^** | **Unadjusted OR**  **(95% CI)** | **Adjusted OR**  **(95% CI) ^b^** |
| --- | --- | --- | --- | --- |
| **Cross-sectional analysis** | **First wave appeared in (Wave 2)** | | **Final wave appeared in (Wave 4)** | |
| Non-CALD | Reference | Reference | Reference | Reference |
| CALD | 1.97 (0.90, 4.33) | 1.87 (0.69, 5.07) | No observations | No observations |
| **Longitudinal analysis** | | | | |
| CALD vs non-CALD | 0.93 (0.39, 2.21) | 0.58 (0.12, 2.78) |  | |

^a^ Adjusted for country of birth, access to private health insurance and highest education level

^b^ Adjusted for country of birth, employment status, access to private health insurance and highest education level
